# Sterilization plan of the used metered dose inhalers (MDI) to avoid wastage amid COVID-19 pandemic drug shortage

**DOI:** 10.1186/s40545-020-00224-4

**Published:** 2020-05-20

**Authors:** Ali Elbeddini

**Affiliations:** Chairman of Pharmacy and patient safety officer for Winchester Hospital, Winchester District Memorial Hospital (WDMH), 556 Louise St, Winchester, ON K0C2K0 Canada

**Keywords:** Metered Dse inhalers (MDI), Sterilization, Drug shortage, COVID-19

## Abstract

Background

Coronavirus is causing a shortage of critical inhalers needed by patients with Asthma and respiratory illness. Patients with Asthma are at higher risk if they tract the novel Coronavirus. As the coronavirus continues to spread, hospitals are turning to use more salbutamol MDI. Salbutamol MDI has become the line of defence for physicians in the emergency room who are treating patients with Corona Virus Disease 2019 (COVID-19) and have respiratory distress .[Hui et al 2020 ,and Center for Drug Evaluation and Research 2020]

During the COVID pandemic, there has been a drastic increase in the use of MDI inhalers; therefore, it led to a decrease in availability and a break in the supply chain. Patients with Asthma are at higher risk if they tract the novel Coronavirus, and an inhaler could be a life or death for them. As the coronavirus continues to spread, hospitals are turning to use more salbutamol Metered Dose inhaler (MDI). Salbutamol MDI is now on short supply as the COVID-19 continues to spread. Salbutamol MDI has become the line of defence for physicians in the emergency room who are treating patients with COVID-19 and have respiratory distress. The current shortage of salbutamol MDI could be a result of stockpiling and hoarding of this life-saving inhaler. That had led to a critical shortage of Salbutamol MDI, and even the case shortage continues with some other alternatives such as Ipratropium MDI and even with long-acting B-agonists such as Salmeterol and Formoterol which also starting to have a limitation on ordering these agents.

Coronavirus sparks fear of medication shortage. Coronavirus panic-buying also may have led to a shortage of critical inhalers. We have also got elderly patients with COPD who may need Ventolin MDI and also premature babies who may have caught Respiratory Syncytial Virus (RSV) and need salbutamol MDI to support their lungs have since been compromised, and they rely heavily on Asthma inhalers. Finding a safe and creative strategy is essential during the COVID-19 pandemic.

## Proposed sterilization plan for the used metered dose inhalers (MDI) [1–4]

There is considerable literature regarding the reprocessing and reuse of previously issued Salbutamol MDI. While a debatable practice, several hospitals have successfully implemented related programs .the current MDI canister protocol states that compliance with disinfecting the MDI nozzle is vital [5, 6]. In one case, cultures were taken of the MDI nozzle before and after disinfection with an alcohol prep pad, as well as after treatments were administered. Growth of *Staphylococcus epidermidis* occurred in at least 5% of the cultures with all three types of specimens, including those taken after the nozzle was disinfected with an alcohol prep pad. In another case, the hospital assessed the failure to wipe the canister nozzle with an alcohol prep pad before patient use; 1 of 18 (5.5%) cultures resulted in the growth of Streptococci Group D (Enterococci) [7]. A discussion may need to take place in each institution that includes Doctors, infection control, and pharmacy to agree on the best way to implement and ensure safety.

**Fig. 1:**
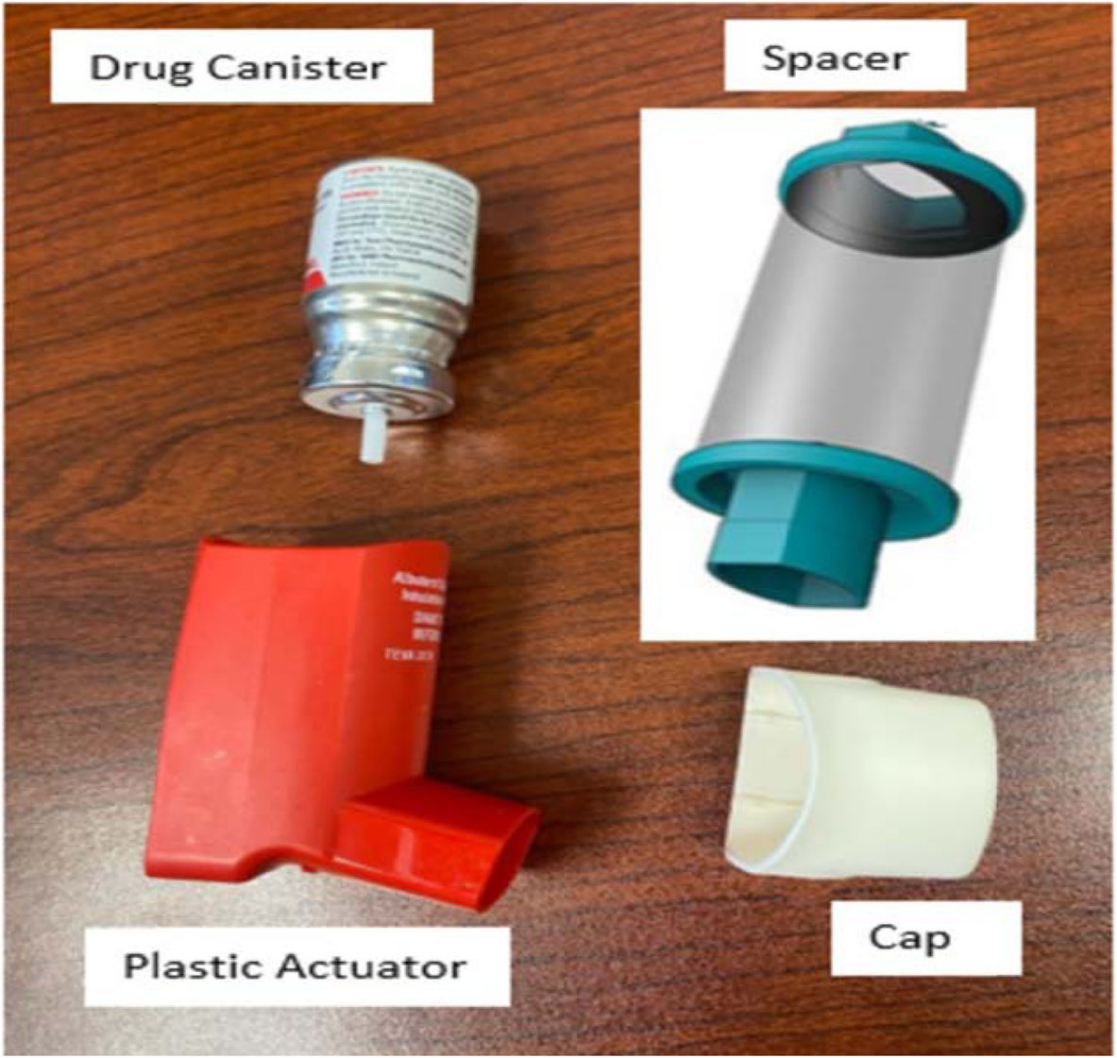
Metered-dose inhaler with a spacer

**Table 1:** **Step by step approach to the sterilization of Metered-Dose inhaler (MDI) (5-7)**

**Steps:** 1. The MDIs must be used with an aerochamber in order for reprocessing to be utilized 2. MDI used on the non-immunosuppressed patient will be recycled once the patient is discharged. 3. When dispensing an MDI, the pharmacy member will heat seal the inhaler (Canister, plastic actuator, and cap) in a plastic baggie with the patient label on the baggie. It is important not to place a label on any part of the inhaler. The pharmacy member will place the sealed baggie containing the inhaler along with one spacer in a plastic bag to be sent to the nursing unit. The outermost plastic bag will be labelled with a sticker to help remind staff of key steps. 4. The inhaler (drug canister + plastic actuator + cap) and the spacer will be kept in the plastic bag in the patient’s room at all times. Upon transfer, all parts of the inhaler and spacer will be transferred with the patient for continued storage and use for the patient. 5. Upon discontinuation of the Patient or Medication, Registered Nurse (RN) or Respiratory Therapist (RT) will ensure all pieces, including drug canister + plastic actuator + cap + spacer, are contained in the patient-specific dispensing bag sent from the pharmacy. RN or RT member will discard the patient label (on the plastic baggie) in the Shred-It bin. 6. (RN) or (RT) will treat all contents as biohazardous and place the bag in the designated bin on each unit. 7. PPE team members will collect MDIs from the bin and deliver to sterile processing for reprocess cleaning and disinfection. PPE team members will wipe the bin following the removal of items each day. 8. The sterile processing department (SPD) personnel will don gloves as the appropriate PPE for disinfecting the inhalers. 9. The spacer will be discarded as a biohazard. 10. The canister of the drug, the plastic actuator with the mouthpiece, and the cap will be wiped thoroughly with 70% isopropyl alcohol: o Remove drug canister from the plastic actuator. o Wipe all surfaces, interior and exterior, with alcohol. o Do not submerge the canister or nozzle in liquid as this can block the nozzle. o Ensure adequate dry time to allow for disinfection. 11. Once dry, place the canister inside a new zip lock bag and sealed. 12. Soak the actuator and mouthpiece cap in Valsure® neutral pH detergent and water for 10 min. 13. Ensure the appropriate dilution rate is followed. Use a timer. Contact time of 10 min is vital to effective disinfection. 14. Use a nylon brush to scrub away any visible soiling or medication build up in and on the Actuator and Mouthpiece 15. Place inhaler components, not the canister, inside the Anesthetic washer manifold and place in Automated Washer/Disinfector. o Select the Anesthetic Cycle. o Once completed, place the components in the HEPA dryer. o Once components are dry and cool, place in a new zip-lock bag labelled PHARMACY 16. SPD will deliver the reprocessed MDIs to Pharmacy to be dispensed for patients 17. Once the inhaler returns to the pharmacy, the inhaler will be quarantined for an additional five days. The release date will be marked on the bag 18. Weigh canister, return to stock for inhalers, according to the table below. 19. Place canister into actuator and mouthpiece and return to a separate inventory.		
Inhaler	Remaining doses	Mean weight (Canister only)
Ipratropium (Atrovent®) *	200 doses	26.289 g ± 2.629
	0 doses	13.842 g ± 1.384
Salbutamol (Ventolin®)	200 doses	28.863 g ± 2.886
	0 doses	12.843 g ± 1.284

*Ipratropium: discard inhaler when weight reaches 18 g*

## Summary

Nebulized medication therapy has been the standard of care for bronchodilation for respiratory patients. However, nebulization generates aerosol, which increases the risk of droplet contamination as droplets can remain in the air and can spread virus particles. Critical inhaler medication shortage looms as coronavirus cases soar.

The use of metered-dose inhalers (MDI) reduces the risk of aerosol-generating particles. During the COVID pandemic, there has been a drastic increase in the use of MDI inhalers, which has led to a decrease in availability and a break in the supply chain. Hospital teams need to be proactive and start collecting the used MDI when appropriate and sterilize it following the provided procedure and keep in separate stock in case it is required. To minimize the risk of contamination, a discussion may need to take place in each institution that includes Doctors, infection control, SPD team and pharmacy to agree on the best way to implement and ensure safety (Fig. 1) (Table 1).

## Data Availability

Data sharing does not apply to this article as no datasets were generated or analyzed during the current study.
